# Side-to-Side Flipping Wedge Osteotomy: Virtual Surgical Planning Suggested an Innovative One-Stage Procedure for Aligning Both Knees in “Windswept Deformity”

**DOI:** 10.3390/jpm13111538

**Published:** 2023-10-26

**Authors:** Grazia Chiara Menozzi, Alessandro Depaoli, Marco Ramella, Giulia Alessandri, Leonardo Frizziero, Alfredo Liverani, Gino Rocca, Giovanni Trisolino

**Affiliations:** 1 Unit of Pediatric Orthopedics and Traumatology, IRCCS Istituto Ortopedico Rizzoli, 40136 Bologna, Italy; graziachiara.menozzi@ior.it (G.C.M.); alessandro.depaoli@ior.it (A.D.); marco.ramella@ior.it (M.R.); gino.rocca@ior.it (G.R.); 2 Department of Industrial Engineering, Alma Mater Studiorum University of Bologna, 40136 Bologna, Italy; giulia.alessandri5@unibo.it (G.A.); leonardo.frizziero@unibo.it (L.F.); alfredo.liverani@unibo.it (A.L.)

**Keywords:** VSP, 3D printing, in-hospital, point-of-care, patient-specific instruments, cutting guide, autograft, windswept deformity, pediatric

## Abstract

(1) Background: The adoption of Virtual Surgical Planning (VSP) and 3D technologies is rapidly growing within the field of orthopedic surgery, opening the door to highly innovative and individually tailored surgical techniques. We present an innovative correction approach successfully used in a child affected by “windswept deformity” of the knees. (2) Methods: We report a case involving a child diagnosed with “windswept deformity” of the knees. This condition was successfully addressed through a one-stage bilateral osteotomy of the distal femur. Notably, the wedge removed from the valgus side was flipped and employed on the varus side to achieve the correction of both knees simultaneously. The surgical technique was entirely conceptualized, simulated, and planned in a virtual environment. Customized cutting guides and bony models were produced at an in-hospital 3D printing point of care and used during the operation. (3) Results: The surgery was carried out according to the VSP, resulting in favorable outcomes. We achieved good corrections of the angular deformity with an absolute difference from the planned correction of 2° on the right side and 1° on the left side. Moreover, this precision not only improved surgical outcomes but also reduced the procedure’s duration and overall cost, highlighting the efficiency of our approach. (4) Conclusions: The integration of VSP and 3D printing into the surgical treatment of rare limb anomalies not only deepens our understanding of these deformities but also opens the door to the development of innovative, personalized, and adaptable approaches for addressing these unique conditions.

## 1. Introduction

The adoption of 3D technologies is increasingly becoming the standard for treating several orthopedic conditions [1]. There is convergent evidence that tools such as Virtual Surgical Planning (VSP) and 3D Printing offer numerous advantages in orthopedic surgery by reducing surgical time, use of fluoroscopy, risk of infection and bleeding, and increasing surgical precision [2,3,4,5,6,7]. VSP plays a pivotal role in corrective surgery for skeletal deformities, especially when these deformities exhibit multiplanar, multifocal, or multi-segmental characteristics. In such cases, VSP and 3D printing provide both physical and virtual 3D models, enabling an in-depth study of the deformity and optimizing correction strategies, including novel surgical approaches. In this scenario, VSP allows for realistic surgical simulation and rendering of the desired correction, advancing the capacity to conceive, plan, and execute groundbreaking surgical procedures never attempted before. The concomitant use of 3D-printed templates and cutting guides increases the precision, safety, and efficacy of these procedures, significantly reducing the learning curve and the risk of errors.

In this study, we present a case of “windswept deformity” of the knees in a 15-year-old boy. “Windswept deformity” is defined as the phenotypical presentation of a varus and valgus deformity with variable localization and underlying pathologies [8]. A recent systematic review identified 184 cases of children with “windswept deformity” reported in the literature in patients affected by rickets or skeletal dysplasias in the majority of cases [8]. Considering the rarity of this deformity and the wide range of potential underlying causes, there is currently a lack of evidence-based guidelines for surgical treatment. Depending on factors such as the patient’s age and the severity of the deformity, various approaches have been described by different authors. These approaches may include acute correction techniques, gradual correction methods, or guided growth procedures [8].

The patient was treated with a single-stage acute correction with bilateral osteotomy. A medial closing-wedge distal femoral osteotomy was performed on the right side, and a medial opening-wedge distal femoral osteotomy was carried out on the left side. Notably, the wedge removed from the valgus side was shaped, flipped, and employed as a cortico-cancellous massive autograft on the varus side. To enhance precision, the procedure was meticulously planned and simulated in a virtual environment. We utilized 3D-printed templates and cutting guides to ensure precise correction and to determine the appropriate size, positioning, and orientation of the screw plates [2]. The entire process was performed in an in-hospital low-cost 3D printing Point-Of-Care (POC) [2,5,6,7,9].

This report aims to illustrate that VSP serves not only as an essential tool for achieving precise corrections but also as a valuable resource for streamlining surgical procedures, innovating techniques, and promoting more judicious bone graft utilization [9].

## 2. Materials and Methods

### 2.1. Case Presentation

**Figure 1:**
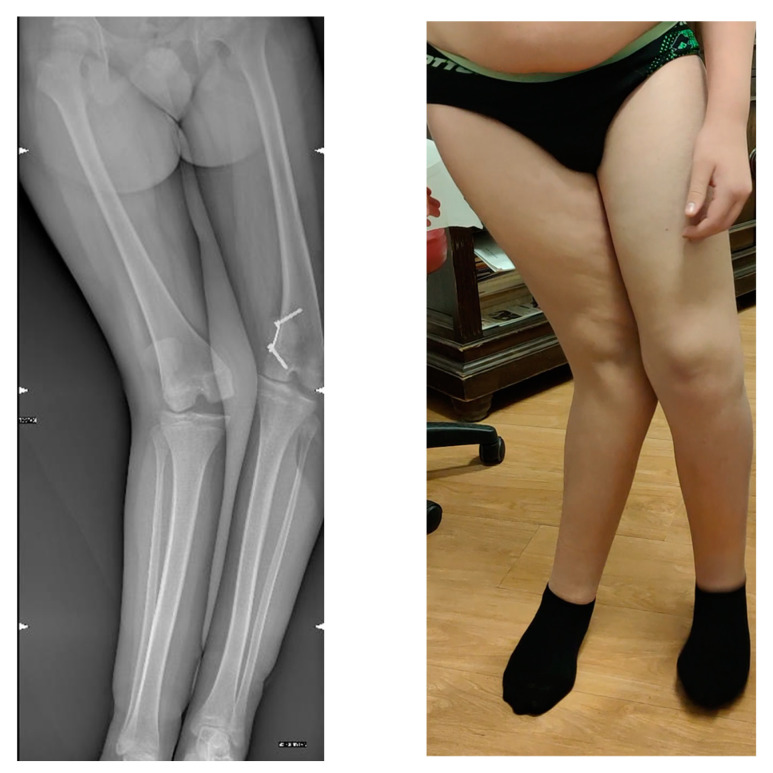
Radiographic and clinical aspects of the limbs.

We present a case of a 15-year-old male patient affected by a “windswept deformity” of the knee. Since infancy, he presented a global developmental delay without any recognizable syndrome. Between age 5 and 8, he suffered bilateral “Perthes’-like” disease, developing residual deformity and shortening of the left femur. At age 10, the patient developed a bilateral valgus knee, which was treated elsewhere with bilateral hemiephysiodesis with tension band plates (TBPs). At age 11, the TBP on the right side was removed. At age 15, he came to our attention with valgus alignment of the right knee and varus alignment of the left knee (Figure 1). The left femur was 4 cm shorter (92% of the controlateral). The patient also showed multidirectional instability of both knees due to generalized hyperlaxity. At the preoperative assessment, the patient’s height measured 158 cm, and his weight was 62 kg, resulting in a BMI of 24.8, which falls within the 89th percentile for age and gender. During the stance phase, the patient displayed approximately 25° of clinical valgus deviation on the right side and approximately 20° of clinical varus deviation on the left side. During walking, there was no significant dysbasia of the pelvis.

### 2.2. Image Acquisition and 3D Model Reconstruction

A full lower limb CT scan, spanning from the pelvis to the feet, was conducted using a low-dose protocol already described elsewhere [6,7,9]. The resulting CT images were transformed into 3D digital models through a segmentation process. The entire protocol of computer-aided segmentation, reconstruction, simulation, and VSP has already been described.

Typically, we examine both limbs in CT scans for limb deformities to establish a personalized basis for correction by mirroring the unaffected side. Unfortunately, in this case, a contralateral comparison was not feasible. At this juncture, the surgeon proposed simultaneously correcting both knees. This involved a medial closing-wedge varus osteotomy of the distal femur on the right side and an open wedge valgus osteotomy of the distal femur on the left side, with the wedge removed from the right being transferred to the left osteotomy. Extensive literature research had not uncovered any previous instances of a similar intervention, making it essential to conduct a simulation in a virtual environment to assess the feasibility of this novel procedure.

**Figure 2:**
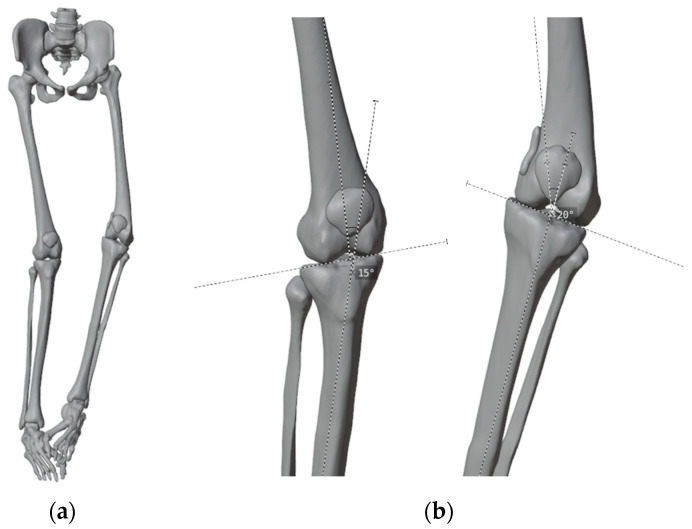
(**a**) 3D virtual model obtained from CT scan; (**b**) The calculated angles of deformity on the 3D model.

Once the 3D virtual model was completed, we compared the degree of deformity as assessed on the coronal plane of long-standing radiographs of the lower limbs with the analysis of the 3D virtual representation. This comparison allowed us to verify the alignment and gain a better understanding of the extent of deformity in three-dimensional space (Figure 2).

### 2.3. Surgical Simulation and Planning

Once the skeletal reconstruction of the deformity was completed, the 3D model was used to simulate the corrections. Some surgical treatment options were simulated to understand which correction would have been the most suitable.

To correct both angular deformities and leg length discrepancy, we planned a shortening osteotomy of the right femur and an opening wedge osteotomy of the left femur using a portion of the resected bone as an autograft on the contralateral side [10].

**Figure 3:**
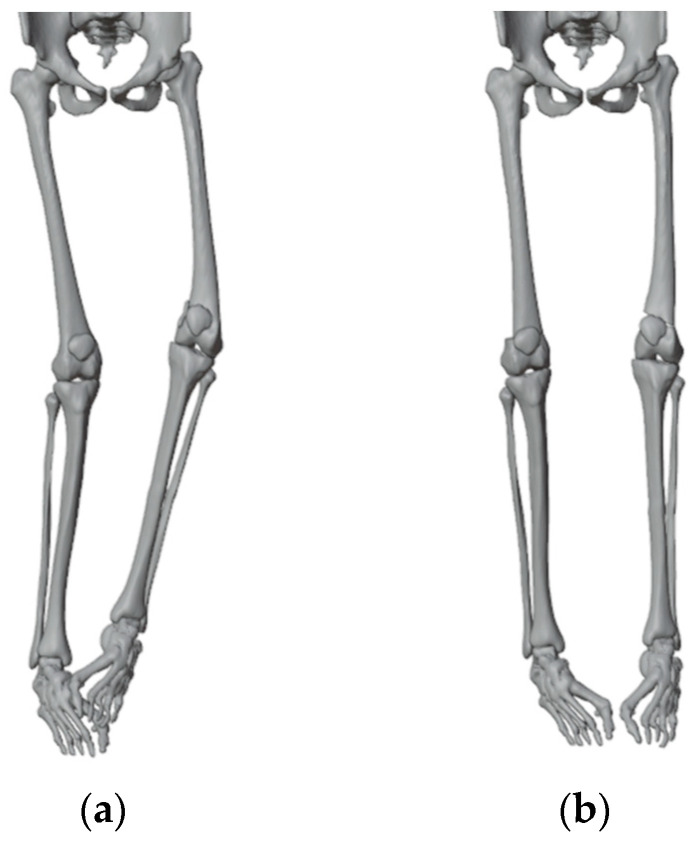
(**a**) Before the simulation of the corrections; (**b**) After the simulation of 15°of varus correction on the right and 20° of valgus correction on the left.

We simulated some surgical plannings to visualize the most suitable angulation to achieve a good correction: 15 degrees of correction for the right femur and 20 for the left (Figure 3). While we did observe a remaining obliquity of the knee joint line after the simulated correction, we made the decision not to perform concurrent tibial correction to minimize both the number of procedures and their extent. We also accepted the medial translation of the femoral shaft by 12 mm relative to the distal metaphysis.

**Figure 4:**
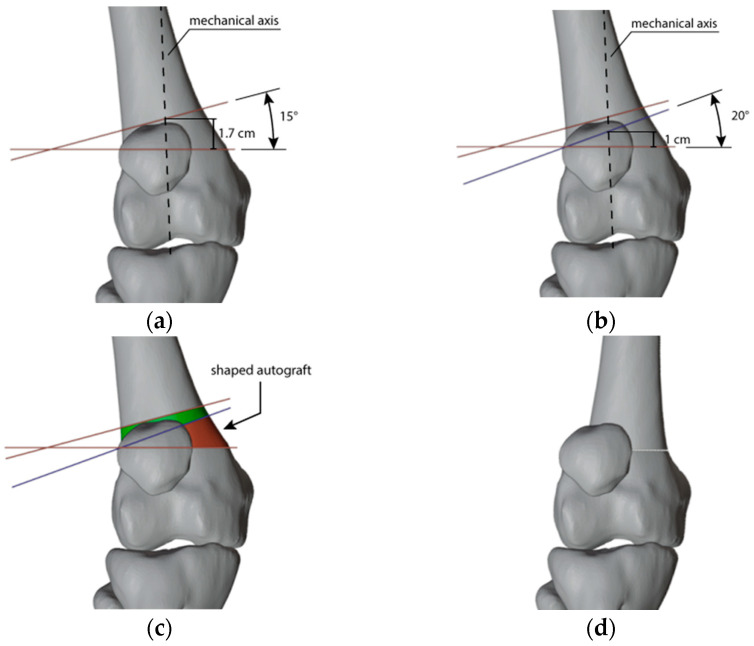
(**a**) Representation of the cuts (in red) to correct the right femur; (**b**) The cut (in blue) to shape the autograft to be used in the left-sided correction; (**c**) The resulting autograft (in red); (**d**) The resulting correction on the right femur.

For the right femur, the plan involved removing a trapezoidal bone wedge with a 15° angle between the cutting planes. This resulted in a shortening of 1.7 cm, calculated by accounting for the wedge’s thickness around the transition of the mechanical axis, reducing the leg length difference from 4 cm to 2.3 cm. The graft harvested from the right side was shaped and flipped to provide the massive autograft for the 20° opening wedge correction on the opposite side. Additionally, the height of the wedge obtained, calculated in the region where the mechanical axis transitioned, closely matched the thickness of the central portion of the wedge itself, resulting in a further elongation of approximately 1 cm on the shorter side (Figure 4). As a result, the initial limb length discrepancy decreased from 4 cm to 1.3 cm.

**Figure 5:**
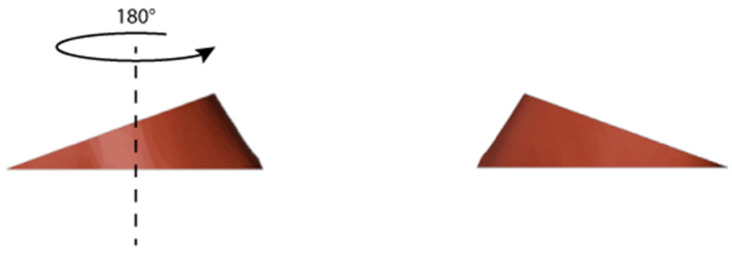
To be implanted, the resulting autograft had to be flipped by 180 degrees on its axis.

**Figure 6:**
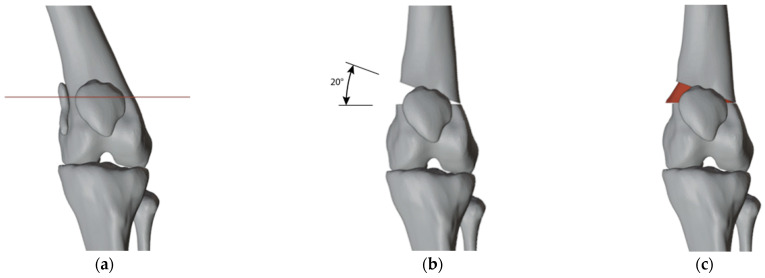
(**a**) The cut on the left side to perform the opening wedge osteotomy; (**b**) The 20 degrees varus correction; (**c**) Insertion of the autologous bone graft in the correct position.

**Figure 7:**
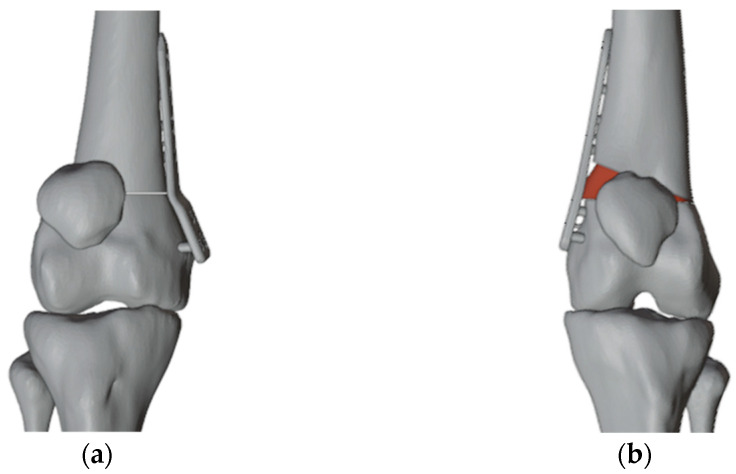
(**a**) The plate chosen for the right femur; (**b**) The plate chosen for the left femur.

The autograft was implanted by rotating it 180 degrees on its axis for a more precise fit within the insertion site (Figure 5 and Figure 6). Furthermore, we determined the appropriate plates to use by selecting those that most closely matched the patient’s anatomical structure (Figure 7).

### 2.4. Design and Production of the Patient-Specific Instrumentation

We designed Patient-Specific Instrumentation (PSI) to support the surgery. We realized three PSIs for the right femur and one for the left. All PSIs were made by tracing the patient’s anatomy at the point of support to ensure good positioning.

#### 2.4.1. PSIs Design for the Right Femur

**Figure 8:**
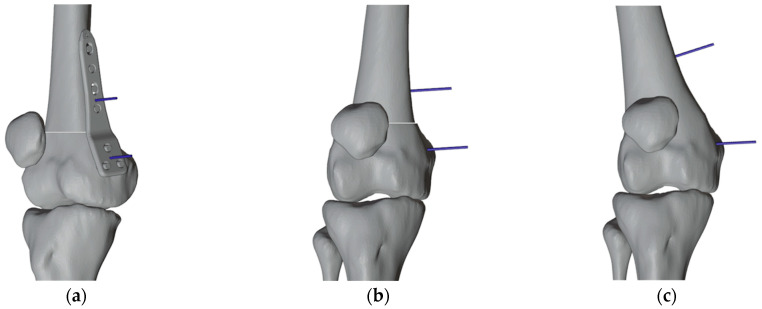
Reverse planning method to identify the plate pins positioning in the right femur. (**a**) The corrective osteotomy was simulated; the plate was applied on the medial surface of the distal femur, and reference pins of the plate were positioned accordingly (antero-medial view); (**b**) the virtual plate was removed (anterior view); (**c**) The distal femur was then returned to its initial condition while keeping the reference pins in place.

**Figure 9:**
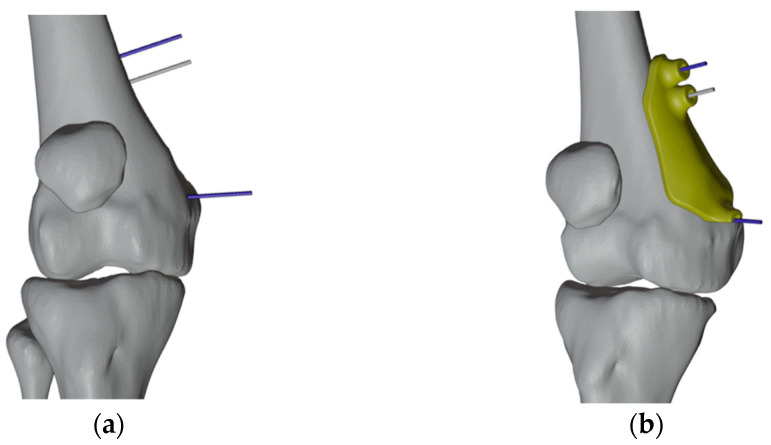
(**a**) Reference plate pin (blue) and additional pin (grey); (**b**) The first PSI with the distal slot open.

The first PSI was created to set the reference pins. The initial pin placement was determined by applying the principles of the “reverse planning method,” which involves beginning with the desired final correction and working backward to the preexisting deformity (Figure 8). We decided to insert an additional proximal wire to decrease the degrees of freedom for subsequent cutting guides. Due to the initial different directions of the wires, the distal wire slot was made partially open to allow easy removal of the first mask once the wires were inserted (Figure 9).

**Figure 10:**
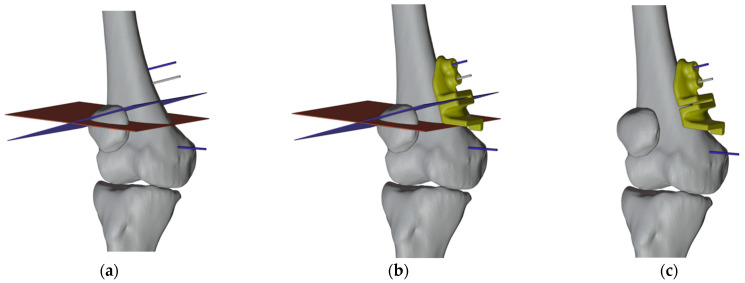
(**a**) Visualization of the chosen cutting planes and pins; (**b**) Design of the PSI with slots corresponding to pins and cuts; (**c**) PSI placed.

The second PSI was designed with two proximal wire slots to be placed correctly and two cuts slot to harvest the autologous graft for the valgus correction of the left femur (Figure 10).

**Figure 11:**
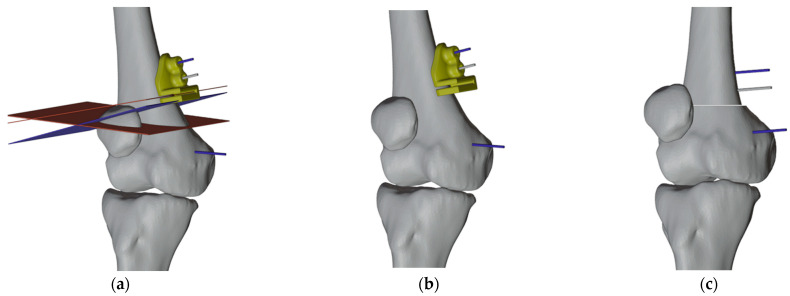
(**a**) Design of the PSI with slots corresponding to pins and to the last cut; (**b**) PSI placed; (**c**) Pins after the corrections.

The third PSI was realized with the two proximal wire slots and one cut slot to complete the planned resection and to achieve the 15° varus correction (Figure 11).

#### 2.4.2. PSI Design for the Left Femur

**Figure 12:**
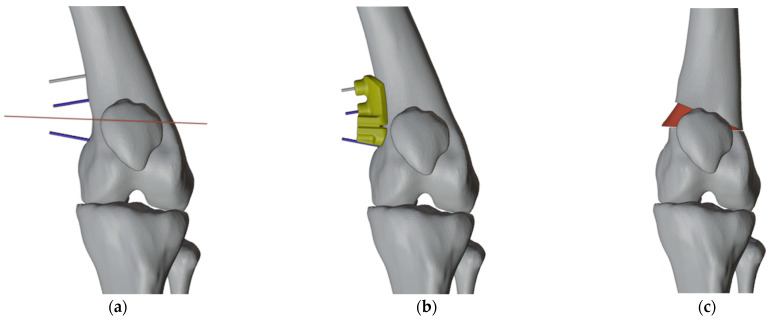
(**a**) Visualization of the chosen cutting planes and pins; (**b**) Design of the PSI with slots corresponding to pins and cuts; (**c**) Final correction with parallel pins.

Only one PSI was made for the left femur. Again, the “reverse planning method” was applied, and a proximal pin was added to provide rotational stability when performing the osteotomy. The cutting guide had two parallel proximal pin slots, an open slot for the distal pin that had a different direction, and a slot to identify the position of the opening wedge osteotomy. After inserting the autologous bone graft from the right femur and completing the corrections, the reference pins were realigned to guide the placement of the plate (Figure 12).

#### 2.4.3. 3D Printing of PSI and Sterilization

**Figure 13:**
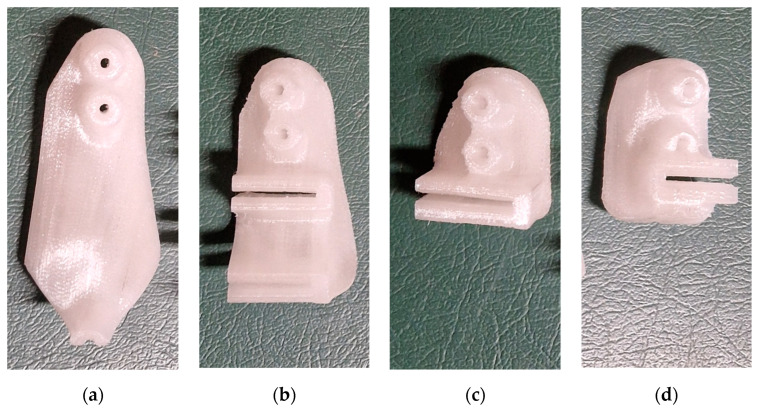
3D printed cutting guides treated and ready for sterilization: (**a**–**c**) First, Second, and Third PSI for the right femur; (**d**) PSI for the left femur.

All PSIs were subsequently 3D printed with Qidi I-Mates S, with a layer thickness of 0.2 mm and a printing accuracy of 0.05–0.2 mm, and heat treated to be sterilized, according to a well-described procedure [11] (Figure 13). On the day of the surgery, the PSIs were delivered sterilized directly to the operating room along with operative instructions and standard instruments.

### 2.5. Surgical Treatment

**Figure 14:**
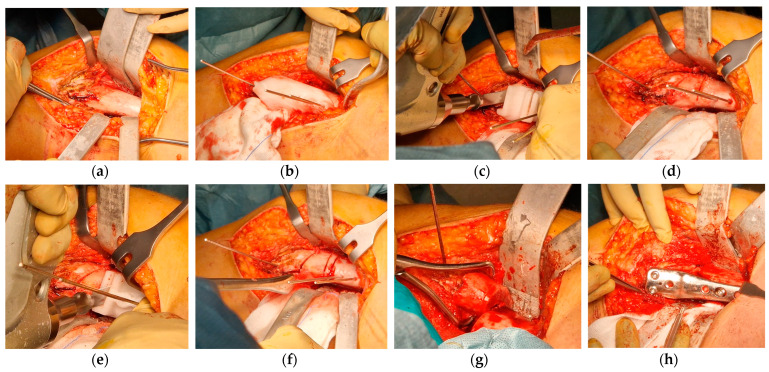
Steps of the surgical procedure on the right knee: (**a**) distal metaphysis of the right femur was exposed through a medial approach; (**b**) the first PSI was positioned by finding the best fit on the bone shape to guide the insertion of the pins (one distal and two proximal); (**c**) the second PSI was stabilized with the two proximal pins to find the cutting planes of the bone autograft; (**d**) incomplete cuts were visible at the osteotomy site; (**e**) the third PSI was positioned with the proximal pins to find the most proximal cutting plane; (**f**) the three incomplete cutting planes were identified, the distal and the proximal ones were completed to perform the shortening and varus osteotomy; (**g**) the trapezoid wedge was removed, the inner cut was still visible, and it was completed on a sterile surgical trolley; (**h**) final fixation with an LCP plate.

The patient was placed in a supine position. The right leg was included in the first surgical field with the tourniquet. A 12–15 cm longitudinal incision on the medial side of the distal right femur was performed, and through a sub-vastus approach, the distal femur was exposed. Before performing any cut, a longitudinal mark on the cortical bone was made with the saw for rotational reference. Shortening osteotomy and angular correction were achieved as planned, and the bone was fixed with a locking-compression plate (LCP) DFOS 4.5 mm, 90°, 4-holes, offset 4 mm (OrthoPediatrics, Warsaw, IN, USA). The wedge that was removed was protected in wet, sterile gauze. The medial collateral ligament was tensioned with absorbable 1 Vicryl sutures to increase articular stability. The tourniquet was released, and a suture was performed, leaving a drainage (Figure 14).

**Figure 15:**
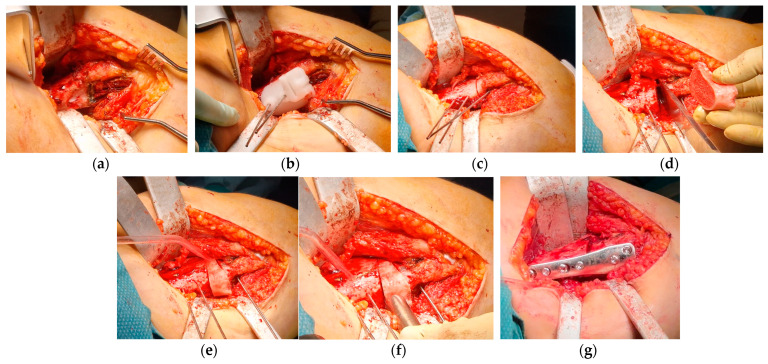
Pictures of the surgical procedure on the left knee: (**a**) distal metaphysis of the left femur was exposed through a medial approach, and the tension band plate was removed (dark spot); (**b**) the PSI was positioned by finding the best fit on the bone to perform incomplete opening wedge osteotomy; (**c**) the cut for the opening wedge osteotomy was visible on the bone; (**d**) with a chisel, the cortical cut was gradually extended in order to fit the autograft from the contralateral shortening osteotomy (on the right) maintaining a bone hinge on the lateral cortex; (**e**,**f**) the autograft was positioned and impacted to achieve the planned angular correction; (**g**) final fixation with an LCP plate.

A second surgical field was prepared, including the left leg, with a tourniquet applied. A 12–15 cm longitudinal on the medial side of the left femur was performed, and through a sub-vastus approach, TBP was identified and removed. According to the VSP, an opening wedge osteotomy was performed with a saw and chisels, preserving a lateral bone hinge. As planned, a portion of the bone removed from the right femur was used as an autograft, and the osteotomy was fixed with a locking-compression plate (LCP) DFOS 4.5 mm, 90°, 4-holes, no offset (OrthoPediatrics, Warsaw, IN, USA). Despite the osteotomy, tensioning of the medial collateral ligament was also performed in the left knee to increase articular stability. The tourniquet was released, and a suture was performed, leaving a second drainage (Figure 15).

## 3. Results

**Figure 16:**
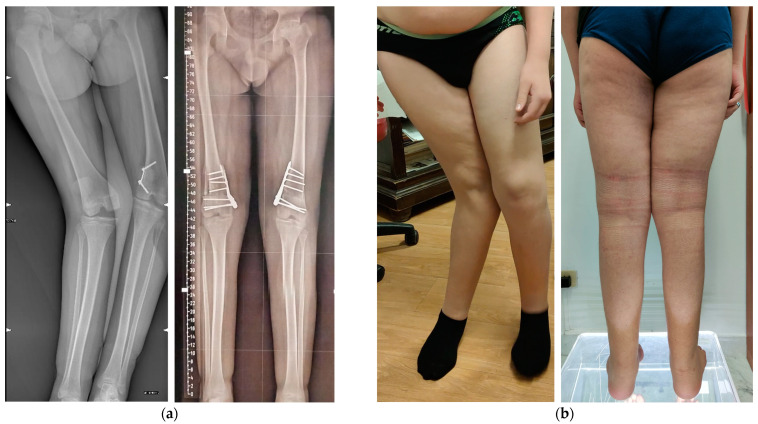
(**a**) Preoperative and postoperative (+6 month) radiographs; (**b**) Clinical pictures before and after surgery.

**Figure 17:**
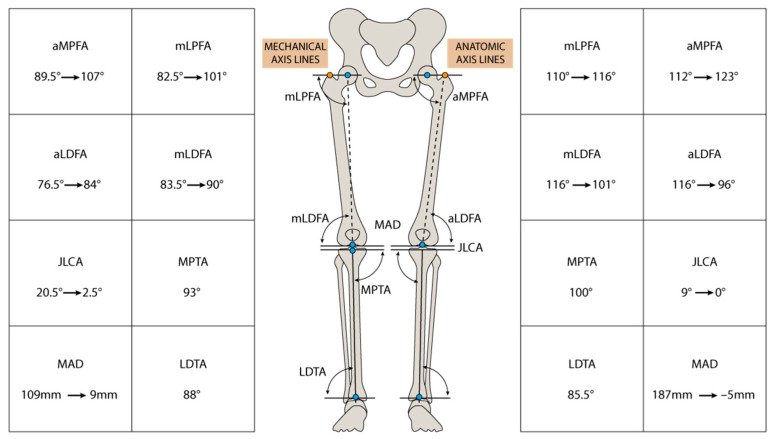
Preoperative and postoperative angles between articular lines and both mechanical and anatomical axes. aMPFA: anatomical Medial Proximal Femur Angle; aLDFA: anatomical Lateral Distal Femur Angle; JLCA: Joint Line Convergence Angle; MAD: Mechanical Axis Deviation; mLPFA: mechanical Lateral Proximal femur Angle; mLDFA: mechanical Lateral Distal Femur Angle; MPTA: Medial Proximal Tibial Angle; LDTA: lateral Distal Tibial Angle.

The surgical procedure proceeded smoothly without notable hitches or intraoperative complications. Overall, 11 intraoperative fluoroscopy shots were required (5 for the right femur and 6 for the left femur), with a total fluoroscopy time of 5 s and median dose-area product (DAP) of 8.10 cGycm^2^. After surgery, both knees were immobilized with extension braces for 30 days. Two days after the surgical procedure, the patient required a transfusion of one unit of RBC. Full weight-bearing was gradually allowed on the right side, while partial weight-bearing was maintained for 8 weeks on the left knee to promote bone integration of the graft. At the latest follow-up one year later, the patient could walk without support, with satisfactory clinical and radiographic alignment of the lower limbs (Figure 16 and Figure 17). Angular correction was assessed on the radiographs taken at the last available follow-up by comparing the angular values with the preoperative radiographs and relating the achieved correction to the planned correction by VSP (see Table 1). Radiographs showed complete bone healing of the osteotomies and osteointegration of the bone graft. The residual LLD was 1 cm (left limb shorter). However, he was recommended to use soft knee braces with lateral stabilizers to reduce residual knee joint instability.

## 4. Discussion

This case report described how VSP and 3D printing can be effectively used for simulating and planning an innovative method for correcting the windswept deformity of the knees, ensuring feasibility and safety for the patient. To the best of our knowledge, this is the first case of windswept deformity in a child treated by simultaneous corrective osteotomy of both knees.

In literature, “windswept deformity of lower limbs” may be referred to as an unusual condition typically seen in non-ambulatory children with cerebral palsy [12]. In such cases, the deformity often stems from an underlying condition involving asymmetric contracture of the hips. However, in ambulatory children, this deformity mainly affects the knees and is also referred to as varo-valga knees. A recent systematic review revealed that there were fewer than 200 documented non-neurologic pediatric cases, typically attributed to causes such as rickets, metabolic disorders, genetic skeletal dysplasias, or trauma [8]. Nevertheless, the etiology remains unknown in most cases.

Most of the studies about surgical treatment of windswept deformity have been focused on adults and total knee replacement [13,14,15], with limited attention given to surgical interventions for children and young adults with this condition. However, two systematic reviews on knee replacement in windswept deformity concur that simultaneous knee replacement benefits patients, surgeons, and the healthcare system. Babu additionally recommends using resected bone from the less affected side as a graft for the other side [16,17].

Poor information has been found in the literature concerning the surgical treatment of windswept deformities in children and young adults.

Some authors reported data about children with windswept deformity treated by emiepiphysiodesis. Gigante et al. reported data from seven children with renal osteodystrophy treated by hemiepiphysiodesis around the knee. Among them, only one child exhibited windswept deformity, necessitating multiple surgical procedures to achieve a satisfactory knee alignment [18]. Castaneda et al. reported one case of windswept deformity in a group of 48 patients who underwent emiepiphysiodesis. However, they did not provide any specific information regarding the outcome of the surgical treatment for this patient.

Dudkiewicz documented a case of windswept deformity in an 18-year-old boy with hypophosphatemic rickets who underwent treatment using a Wagner external fixator. This case was complicated by the patient’s limited tolerance to the frame due to severe, unmanageable pain, significant restrictions in knee motion, and the recurrence of the deformity following frame removal. Consequently, the patient needed an additional staged double osteotomy procedure three years later to attain the desired correction.

Eralp et al. described two cases with windswept deformities, successfully corrected by using a fixator-assisted intramedullary nailing technique [19]. While the authors reported satisfactory outcomes, they emphasized that mastering this technique presents a steep learning curve. Furthermore, they underscored the importance of mandatory high-quality intraoperative radiographs as a crucial factor for ensuring precision.

In our case, the decision to perform osteotomies on both femurs simultaneously undoubtedly benefited from the extensive use of surgical simulation, VSP, and 3D-printed PSIs. The accuracy of the angular correction, which is the sole parameter we were able to assess through postoperative radiographs, closely aligns with findings in the existing literature. In fact, previous studies have reported a range of precision values, including 0.3° ± 2.1° and 1° ± 0.9°, and our results are consistent with these benchmarks [20,21]. Moreover, the use of PSI resulted in significant savings in surgical time and blood loss and eliminated the mandatory need for high-quality intraoperative radiographs. Additionally, this has allowed us to beneficially utilize the wedge removed from one side as a graft on the other side, resulting in a further reduction of the risks associated with homologous or heterologous grafts, along with their costs. Even though the procedure was carried out by a surgeon with over 20 years of experience, it was observed that surgical simulation, planning, and 3D printing could reduce the steep learning curve required for performing these interventions. The possibility of conducting the entire process through “in-house 3D printing point-of-care” offers an additional advantage in cost reduction, allowing synergistic work between surgeons and designers, enabling high quality, low cost, and speed of simulation and production [22].

There are additional concerns that warrant consideration in this case.

First, our main objective was to rectify the windswept deformity through femoral osteotomy on both sides. Although we successfully achieved a satisfactory knee alignment on the frontal plane, this resulted in an oblique joint space due to the residual valgus deformity of the tibial plateau. We intentionally selected this approach to avoid additional varus osteotomies on both the tibias. Nonetheless, we are uncertain whether this oblique joint alignment could potentially pose future complications. While in vitro studies have suggested a potential link between an oblique joint line in the frontal plane and medial-lateral knee subluxation, this relationship has not been confirmed in vivo, and there are currently no established pathological threshold values for joint line obliquity [23,24,25,26].

Second, we overlaid the correction calculated from weight-bearing longstanding radiographs of the lower extremities onto a model generated from a supine patient’s CT scan. We opted for this approach due to the unavailability of performing a complete standing pelvic and lower limb CT scan. This approximation may have introduced assessment bias. Unfortunately, our hospital lacks the capability to conduct long-standing X-rays using the EOS method. We anticipate that incorporating information from EOS images into the CT-derived model could provide a better understanding of the three-dimensional impact of body weight on the deformity [27]. Additionally, new CT devices enabling scans with the patient in a standing position are expected to become available in the near future [28].

Third, the surgical simulation process does not yet consider the impact of soft tissues, such as capsules, ligaments, and muscles, on joint stability and the ultimate outcome. Despite performing medial capsuloligamentous retensioning on both knees, it was not enough to fully restore knee joint stability. The patient still shows signs of multidirectional instability, partly due to disease-related muscle weakness, and needs permanent knee braces with semi-rigid lateral support. Further research is needed to incorporate the effect of soft tissue in the simulation process and virtual surgical planning.

## 5. Conclusions

In conclusion, we have introduced an innovative technique to address the windswept deformity, an exceptionally rare pediatric deformity of both knees. Our method heavily relies on computer-aided surgical simulation, VSP, and 3D printing cutting guides tailored to the anatomy and deformities of patients, enabling precise corrections in procedures while reducing the risk of complications but also minimizing the duration of the surgery, leading to faster recovery times for patients and less healthcare expenses. We have confidence in the safety and effectiveness of our VSP process, which opens the door to previously unattainable procedures. More research is crucial to fully understand how accurate these methods can be, especially when using 3D-printed guides. We also need to work on creating dynamic musculoskeletal digital twins and better surgical simulations that consider both the physical structure and functional aspects of the musculoskeletal system. These developments are essential for improving the accuracy and effectiveness of surgical procedures.

## Figures and Tables

**Table 1:** Planned and obtained Angular Corrections.

	Planned AC	Obtained AC	Difference
Right	15°	13°	−2°
Left	20°	19°	−1°

## Data Availability

Data are available from the corresponding author upon reasonable request.
